# RGD-Binding Integrins in Head and Neck Cancers

**DOI:** 10.3390/cancers9060056

**Published:** 2017-05-26

**Authors:** Hanadi Talal Ahmedah, Laurence H. Patterson, Steven D. Shnyder, Helen M. Sheldrake

**Affiliations:** 1 Radiological Sciences Department, College of Health and Rehabilitation Sciences, Princess Nourah Bint Abdulrahman University, Riyadh 11564, Saudi Arabia; HTAhmedah@pnu.edu.sa; 2 Institute of Cancer Therapeutics, University of Bradford, Bradford BD7 1DP, UK; l.h.patterson@bradford.ac.uk (L.H.P.); s.d.shnyder@bradford.ac.uk (S.D.S.)

**Keywords:** integrin αv, β1, β3, β6, arginine-glycine-aspartate (RGD), metastasis, treatment resistance

## Abstract

Alterations in integrin expression and function promote tumour growth, invasion, metastasis and neoangiogenesis. Head and neck cancers are highly vascular tumours with a tendency to metastasise. They express a wide range of integrin receptors. Expression of the αv and β1 subunits has been explored relatively extensively and linked to tumour progression and metastasis. Individual receptors αvβ3 and αvβ5 have proved popular targets for diagnostic and therapeutic agents but lesser studied receptors, such as αvβ6, αvβ8, and β1 subfamily members, also show promise. This review presents the current knowledge of integrin expression and function in squamous cell carcinoma of the head and neck (HNSCC), with a particular focus on the arginine-glycine-aspartate (RGD)-binding integrins, in order to highlight the potential of integrins as targets for personalised tumour-specific identification and therapy.

## 1. Introduction

Head and neck cancer is a term used for any malignancies that originate from the oral cavity, oesophagus, pharynx, larynx, paranasal sinuses and nasal cavity [1]. The most recent analysis (Global Burden of Disease 2015) reported nearly 820,000 deaths from head and neck cancers occurring in 2015 [2,3]. Deaths due to lip and oral cavity cancer have increased by 32.5% over the 10 year period 2005–2015, with most other regions of the head and neck showing increases of 13–24% over the same period [3]. The most common type of head and neck cancer is squamous cell carcinoma of the mucosal surfaces (HNSCC) which accounts for about 90% of all cases [4]. Risk factors for HNSCC include the use of tobacco, alcohol and infection with human papillomavirus (HPV). Changes in incidence of oral cancer have been shown to parallel changes in tobacco use in developed and developing countries, and cases associated with HPV infection are increasing [1].

Surgery is the mainstay of HNSCC treatment, but many patients present with locally advanced disease (characterized by lymph node metastasis) which requires additional radiotherapy and/or chemotherapy [5]. Surgical cure will benefit from predictive biomarkers developed to improve identification of resectable HNSCC and lesions with malignant potential. For advanced disease, radiation treatment concurrent with chemotherapy has been recognized to improve survival [6,7,8,9], but causes severe, long-term side effects [10,11]. There is a need for both HNSCC biomarkers of disease progression and molecular targeted therapies in order to improve outcome of both surgery and overall survival from advanced metastatic disease.

Integrins are heterodimeric transmembrane glycoproteins consisting of an α-subunit and a β subunit [12]. In vertebrates, eighteen different α subunits and eight different β subunits combine to create 24 different heterodimers [13,14]. Different types of integrins are categorized according to which cell surface, extracellular matrix (ECM) component or inflammatory ligand they bind [15]. Vertebrates have four receptor subgroups: laminin receptors (α3β1, α7β1, α6β1 and α6β4), leukocyte-specific integrins (the β2 subfamily plus α4β1, α4β7 and αEβ7), collagen receptors (α1β1, α2β1, α10β1, α11β1) and arginine-glycine-aspartate (RGD) receptors (αvβ1, αvβ3, αvβ5, αvβ6, αvβ8, α5β1, α8β1 and αIIbβ3) which recognise the triplet sequence arginine-glycine-aspartate (RGD) motif found in many ECM proteins such as fibronectin, collagen, vitronectin, osteopontin and thrombospondin [16]. Members of the RGD-binding subfamily are highly significant in angiogenesis [17] and thrombosis, and have been considered some of the most important integrin targets for drug discovery. Anti-integrin drugs which are designed to block the integrin–extracellular matrix interaction have been developed to combat a range of diseases [12,18,19,20,21]. Some of these integrin-targeted drugs, namely abciximab, eptifibatide and tirofiban targeting αIIbβ3 and natalizumab and vedolizumab targeting the α4 subfamily, are on the market. The remainder are still in clinical trials (recently summarized by Prager et al. [22]).

A number of the RGD-recognising integrins, αvβ3, αvβ5, αvβ8 and α5β1 are involved in controlling angiogenesis [23,24,25]. αvβ3, αvβ5 and α5β1 are expressed on activated endothelial cells during normal tissue regeneration and can become aberrantly expressed in cancers [17]. αvβ6 and αvβ8 are normally expressed on epithelial cells, notably in the lungs and, for αvβ8, the brain [26], where it is expressed on vasculature, synapses, glial cells, and dendritic spines [27]. The RGD-binding integrins support angiogenesis through survival signalling controlling cell proliferation [17], and the localisation and activation of transforming growth factor-β (TGF-β) [26,27,28]. In cancers, increased or altered expression of integrins on tumour cells and associated vasculature leads to tumour progression through a wide range of mechanisms, including supporting cell proliferation and tumour angiogenesis as previously mentioned, supporting the epithelial mesenchymal transition [29,30], promoting migration and invasion [31,32,33], interaction with the extracellular microenvironment during the metastatic process [34,35,36], and TGF-β activation facilitating tumour immunosuppression [26,30].

The biological potential and possible approaches to integrin targeting in general have been reviewed by many researchers [18,37,38,39,40,41], however, little attention has been focused on HNSCC. The role of integrins in HNSCC was last reviewed in 2005 [42]; the present review will summarise recent developments in understanding the expression and function of RGD-binding integrins in head and neck cancer progression and metastasis.

## 2. Integrins in HNSCC

Investigation of changes in integrin expression on tumour tissues identifies possible biomarkers for disease progression, and targets for imaging and drug delivery agents. Additionally, linking integrin expression or signalling to tumour growth, dissemination, or response to therapy identifies areas where targeted integrin inhibitors may improve prognosis The expression of RGD-binding integrins in clinical tissue samples is summarized in Table 1. Studies on these receptors will be discussed further in the following sections.

### 2.1. The αv Integrin Subfamily

The αv subunit is present in a number of integrins involved in neoangiogenesis, migration and invasion. Associated with these processes is activation of matrix metalloproteinases (MMPs), and activation of TGF-β. αvβ3 and αvβ5 have been the focus of significant research as anti-angiogenic targets [25,49,50], but αvβ6, αvβ1, and αvβ8 are less well-investigated.

The αv integrin subunit is overexpressed in oesophageal, orbital, eyelid and periorbital squamous cell carcinomas (SCCs), and its expression is associated with tumour invasion and metastasis [51]. High expression of αv integrins promotes proliferation and invasion of oral squamous cell carcinoma (SCC) by activating the mitogen-activated protein kinase/extracellular signal-regulated kinase (MEK/ERK) signalling pathway [52]. The expression of αv integrin subunits is correlated with lymphatic metastasis of laryngeal and hypopharyngeal carcinoma, although the associated integrin β subunit that plays the major role in SCC aggressiveness was not identified [53]. In nasopharyngeal carcinoma, expression levels of integrin αv mRNA and protein are also correlated with tumour size and lymph node spread, and were significantly higher in nasopharyngeal carcinoma tissues than in non-neoplastic inflammation tissues from the same region [54].

αv expression has been shown to be higher in tissues from radioresistant nasopharyngeal carcinomas than in tissues from patients who responded to radiotherapy. In vitro, αv expression in the human nasopharyngeal cell line CNE-2 is upregulated in multi-cellular spheroids compared to standard culture conditions, and blocking αv integrin signaling in the SAPK/JNK pathway increased the radiosensitivity of multi-cellular spheroids in vitro and in vivo [55]. Downregulation of the αv integrin subunit by an antisense oligonucleotide has also been shown to inhibit the proliferation of laryngeal carcinoma cells and enhance their apoptosis [56].

Overall, αv expression appears useful as a biomarker of HNSCC tumour tissue and is amenable for biological targeting as an anticancer strategy. The development of small molecule drugs against αv subfamily integrins is feasible, but targeting a particular subfamily member has so far been a more attractive strategy to the pharmaceutical industry [40]. This requires the biological functions of individual αv subfamily members to be understood.

#### 2.1.1. αvβ3 and αvβ5 Integrins

αvβ3 and αvβ5 are the most thoroughly studied of the αv subfamily. αvβ5 integrin expression has been found to be significantly higher in oral tumour cells and tumour stroma than in control samples of normal oral cavity squamous epithelium [43], and significantly higher in larynx and tongue SCC cells than in epithelium cells in normal tissues. Its expression is associated with lymphatic metastasis and angiogenesis [45,57]. αvβ3 was extensively expressed in endothelia with statistically significant differences between tumour and normal tissue [43]. Transfection of tongue squamous carcinoma cells (Cal27) with β3 cDNA increased expression of integrin αvβ5 alongside inducing expression of αvβ3. This increased αvβ3/β5 expression increased cell adhesion to both fibronectin and vitronectin, migration and invasion, and induced resistance to cytotoxic drugs through reducing Src signaling [58]. Knockdown of integrin-linked kinase (ILK) or αvβ3 enhanced drug sensitivity; targeting αvβ5 did not reverse resistance [58].

In contrast, Jones et al. demonstrated that αvβ5 integrin is expressed in both normal and tumour cells of oral squamous cell carcinoma and the expression of αvβ5 was weak or absent in poorly differentiated lesions [44]. In vitro studies have implicated αvβ5 integrin in cancer regression. αvβ5 integrin-deficient cell lines lost the ability for terminal differentiation and grew in an anchorage-independent manner. When αvβ5 integrin expression was restored, the in vitro malignant phenotype was reversed [59]. Transduction of an αv-negative human SCC line (H357) with retroviral vectors encoding αv integrins showed that αvβ5-expressing cells showed enhanced apoptosis (anoikis) and suppression of AKT activity, whereas αv-negative cells and cells expressing αvβ6 did not [60]. Taken together, results from these groups suggest that αvβ5 may not be a suitable target for anti-integrin therapy due to both its role in normal skin biology, and its failure to improve efficacy or reduce resistance when targeted alongside commonly used chemotherapy agents.

Integrin αvβ3 is strongly implicated in tumour angiogenesis [61]. αvβ3 integrin overexpression has been reported on tumour-associated vessels of human carcinomas [62], in angiogenic vasculature of HNSCC mouse xenograft (including patient-derived xenografts) models [63] and in activated endothelial cells [64]. Li et al. found that chemokine receptor 7 (CCR7) and its ligand CCL19 enhance head and neck cancer cell migration and adhesion by activating αvβ3 integrin [46]. Targeting αv and β3 integrins with antibodies partially decreased the effect of the αvβ3 ligand soluble periostin on the proliferation, migration and invasion of HNSCC cells [65].

Multiple preclinical studies have shown that dual αvβ3 and αvβ5 antagonists inhibit tumour angiogenesis and tumour growth [66]. The prototypical antagonist cilengitide is a RGD-derived cyclic peptide that selectively inhibits αvβ3 and αvβ5 integrins. It demonstrated preclinical anti-tumour activity in many malignant tumours such as glioblastoma, prostate [67] and melanoma [68], progressing to clinical trials for a number of advanced cancers. Despite early promise in glioblastoma [69], cilengitide failed to improve overall survival in the phase III CENTRIC study [70] and therefore development ceased in 2015.

In a single patient case report, cilengitide with gemcitabine inhibited the growth of a highly vascularized head and neck tumour and prolonged life for more than one year, encouraging further investigation in HNSCC [71] despite the fact no preclinical studies had been reported in this tumour type. Cilengitide entered the clinic in a phase I/II trial in HNSCC [72,73]. Doses up to 2000 mg/kg were investigated in the phase I part of the trial. The combination with cisplatin, 5-fluorouracil, and cetuximab was well tolerated in patients with recurrent and/or metastatic disease. No dose-limiting or unexpected toxicities were observed and only minor adverse effects were reported [72]. The phase II part compared once or twice weekly administration of 2000 mg cilengitide in combination with cisplatin, 5-fluorouracil, and cetuximab to cisplatin, 5-fluorouracil, and cetuximab alone. Addition of cilengitide did not improve the progression-free survival in this patient population [73], and it was not further investigated.

Subsequently Heiduschka et al. reported that cilengitide in combination with cisplatin increased apoptosis and gave synergistic growth inhibition in three HNSCC cell lines (SCC25, CAL27 and FaDu). Cilengitide in combination with irradiation gave significant inhibition of colony formation [74]. In ex vivo experiments, cilengitide alone had insignificant effect on colony formation from biopsies of HNSCC [75], although the same group later found it did significantly reduce colony formation when cells were seeded on laminin [76]. Combination of cilengitide with cetuximab is effective at reducing colony formation and cytokine release from HNSCC cells, but the two targeted therapeutics did not act synergistically [76]. These results parallel those seen in other tumours i.e., cilengitide appeared promising preclinically but failed to deliver in the clinic. The difference in results observed depending on the culture environment [75,76] suggests care should be taken in the choice of preclinical models used to evaluate new integrin-targeted agents.

The trial investigators suggested that the failure of cilengitide in head and neck cancer is because HNSCC cells, in contrast to tumour endothelium, tend to express low levels of αvβ3, the integrin most tightly bound by cilengitide [43]. αvβ5 inhibition has possible pro-tumourigenic properties in both HNSCC [44,59,60] and glioblastoma [77], thus a combination αvβ3/αvβ5 targeting agent may be self-defeating in these cancers. Tumour expression of related integrins not effectively inhibited by cilengitide may also provide a mechanism for drug resistance and therapeutic failure. Moreover, Becker et al. showed that cilengitide was not distributed from the plasma to other compartments, and was mainly excreted unchanged by the kidney. Its short half-life means tumours are not exposed to therapeutic concentrations for much of the time which may promote tumour growth [78]. It has been proposed that cilengitide’s poor pharmacokinetics has ultimately led to its development being discontinued [70,78] although the observation that the pan-αv antagonist GLPG0187 was not effective as a monotherapy when delivered by continuous infusion suggests other factors, such as combination of integrins targeted, may be significant [79]. The clinical failure of cilengitide makes dual inhibition of αvβ3 and αvβ5 as an antitumour strategy now difficult to justify, although αvβ3-specific agents may be worth investigating. However, inhibitors targeting both the αv subunit, and αvβ3 have been shown to increase the effectiveness of radiotherapy which may be a valuable strategy in HNSCC given the importance of radiotherapy in its treatment.

#### 2.1.2. αvβ6

Multiple studies have found αvβ6 is overexpressed in HNSCC. One study reported overall that 95% of HNSCCs express this integrin on tumour cells, whereas αvβ3 and αvβ5 are found on tumour-associated vasculature and stromal cells, respectively [80]. αvβ6 expression is tumour specific; normal oral mucosal cells do not express the receptor [44,80,81]. Eriksen et al. demonstrated that β1, β4 and β6 integrins were upregulated in carcinomas compared to the adjacent mucosa within formalin-fixed paraffin-embedded pre-irradiation biopsies from 85 patients with head and neck squamous cell carcinomas (HNSCC) [82]. αvβ6 has also been shown to be more highly expressed in primary laryngeal SCC compared to normal epithelium cells [45] and in thyroid cancer versus normal tissue or thyroid adenomas [83]. αvβ6 is a biomarker of lymph node metastasis [83], however there is no significant relationship between expression levels and clinicopathological features of laryngeal SCC [45].

In in vitro studies, αvβ6 appears to be important for cell migration. Inducing expression of the β6 subunit in the poorly invasive human oral SCC9 cell line increased cell migration on fibronectin and invasion through basement membrane by inducing MMP-3, and increased the cell growth in vitro and in vivo [84]. Additionally, it has been shown that αvβ6 protects SCCs from anoikis by activating an AKT survival signal [60].

αvβ6 is expressed at the tips of migrating epithelial sheets during oral mucosal wound healing [85]. It promotes oral SCC invasion by facilitating the epithelial to mesenchymal transition (EMT) [86], and expression of the full-length β6 subunit has been shown to be required for EMT. Cells expressing β6 acquired a fibroblast-like morphology, increased expression of the mesenchymal marker vimentin, and reduced expression of the epithelial markers keratin and E-cadherin, whereas cells expressing a truncated form of β6 lacking the C-terminus kept their epithelial morphology and did not change vimentin or E-cadherin expression [87].

Targeting αvβ6 integrin with an inhibitory peptide significantly inhibits the proliferation of oral squamous cell carcinoma cells [80]. Xue et al. demonstrated that blocking αvβ6 integrin inhibited HSC-3 cell migration and growth in a three-dimensional collagen gel, and retarded tumour growth in vivo. While blocking αvβ6 integrin inhibited cell adhesion to latency-associated peptide of TGF-β1, combination of anti-αvβ6 with anti-α5β1 antibodies was required for complete inhibition of adhesion to fibronectin [88], thereby identifying a possible new dual inhibition strategy for HNSCC treatment. Cooperative use of αvβ1, αvβ6 and α5β1 integrins to adhere and migrate on fibronectin has previously been reported by Koivisto and colleagues [89]. The development of novel selective small molecules binding αvβ1 [90,91], and αvβ6 and/or αvβ8 [92,93] provides the tools required to further investigate the effect of their inhibition in HNSCC and the design of potential new therapeutic and imaging agents.

#### 2.1.3. αvβ8

Expression of the β8 and β1 integrin subunits has been shown to be slightly higher in laryngeal SCC tissue than in normal epithelium [45], confirming an earlier report of the upregulation of αv and β8 [94]. Little is known on the role of αvβ8 in HNSCC. One study has shown that overexpression of αv in oral SCC cells increased β8 level which promoted cell proliferation and MEK/ERK signalling on type I collagen. Reduction of αvβ8 levels by downregulation of integrin β8 decreased focal adhesion kinase (FAK) and MEK/ERK signalling in response to type I collagen, and thus proliferation and invasion [52]. Further study is required to establish αvβ8 as a biomarker or drug target in HNSCC.

### 2.2. The β1 Integrin Subfamily

Many studies have been carried out investigating the association of the β1 integrin subunit with HNSCC. The interpretation of these studies is complicated by the fact that β1 is widely expressed, and partners 12 α subunits to form members of all the integrin subfamilies: receptors for laminins, collagens, and RGD-containing ECM proteins. The β1 subunit has been proposed as a target in several of the studies described below. Development of small molecule drugs would be challenging because of the diverse range of receptors containing β1, and the potential normal tissue toxicity requires further investigation. β1 expression per se is more likely to be important as a biomarker rather than therapeutic target, which would require identification of the specific β1 integrins promoting pathology.

β1 expression has been associated with HNSCC metastasis in multiple models. β1 integrin subunit levels were significantly higher in primary tumours which had metastasised than in non-metastatic tumours and its expression was inversely correlated with overall survival. Reduction in β1 integrin levels reduced lymph node and lung metastasis in vivo and matrigel invasion in vitro by preventing activation of MMP-2 [95]. In oral and oropharyngeal SCC, β1 has been observed in 90% of tumour samples [96]. Expression of β1 enhanced oral SCC cell motility by activating the ERK pathway, and its knockdown reduced the migration activity of OEC-M1 cells and ERK phosphorylation [97].

β1 [98], α6 [99], and α7 [100] have been identified as HNSCC stem cell markers, with β1 [98] being associated with poor response to radiotherapy and increased risk of relapse and metastasis [98] (α7 has similar associations with poor prognosis and metastasis in oesophageal SCC). Moreover, targeting β1 integrin with an inhibitory antibody increased sensitivity to ionizing radiation and delayed the growth of HNSCC cell lines in 3D cultures and in xenografted mice [101], and has been shown to act by impairing repair of radiation-induced DNA double-strand breaks [102,103]. Combining the anti-β1 monoclonal antibody AIIB2 with a poly ADP ribose polymerase (PARP) inhibitor [102] or EGFR inhibitor [103] enhanced cytotoxicity and radiosensitization, providing better in vivo tumour control compared with monotherapy and irradiation in HNSCC models [103]. The combination of β1 and EGFR inhibition is proposed to act by preventing activation of MEK/ERK signalling, bypassing the effect of a single inhibitor [103]. In future, it is likely that combination therapies involving antagonists of a wider range of integrins will be developed to address drug resistance to targeted tyrosine kinase receptor antagonists, although integrin receptor inhibition as a means to promote radiosensitisation effects are more widely applicable in HNSCC treatment.

More details of the β1 integrin-interacting α integrin subunits in HNSCC became available recently. α11 is a biomarker of cancer associated fibroblasts and is overexpressed in HNSCC stroma [104]. α2, α3, α5 and α6 subunits have been shown to be overexpressed experimentally in three-dimensional extracellular matrix grown HNSCC cells [105], and clinically in tumour tissues versus normal tissues using the Oncomine database [105], and in studies of primary oral squamous cell carcinoma [48]. Expression was particularly extensive in invasive or metastatic cases, with expression being significantly associated with tumour invasion and nodal involvement [48]. Inhibition of α3 had the most significant effect in decreasing clonogenic cell survival, promoting apoptosis and enhancing radiosensitivity, while blocking the β1 and α3 subunits resulted in increased cytotoxicity and radiosensitization compared to α3 integrin blocking alone [105]. Related integrin subunits were proposed to be able to compensate for targeting a single subunit [105], strengthening the argument for tumour-specific combination anti-integrin therapies [40].

The remaining α subunit which interacts with β1 to form a RGD-binding integrin is α8. Little is known regarding its role in any cancer. One study has found α8 to be downregulated in laryngeal SCC compared to normal tissue [94].

#### α5β1

Expression of α5 in oesophageal SCC patient samples is significantly correlated with lymph node metastasis, tumour size and poor overall survival of oesophageal SCC patients, and expression increased in cancer cell lines compared to normal oesophageal epithelial lines [106]. Oral SCC has also been shown to express higher levels of α5β1 integrin than normal cells [43,47], and overexpression of α5β1 was reported in many HNSCC cell lines grown in physiological three-dimensional extracellular matrix [105]. α5β1 and α2β1 were shown to be upregulated in caveolin-1 depleted cells, which are associated clinically with distant metastasis [107].

Knockdown of α5 inhibits oesophageal SCC cells growth, migration, and invasion [106]. A small molecule α5β1 antagonist effectively prevented cell migration and invasion, and reduced clonogenic survival [107], suggesting α5β1 as a target to prevent both local progression and distant metastasis in HNSCC. A Phase I trial of the peptide α5β1 antagonist ATN-161 in patients with progressive heavily pretreated solid tumours resulted in stable disease in 6/26 patients [108]. Based on these results, a Phase II clinical trial of ATN-161 in HNSCC was proposed [109] although no further progress has been reported.

### 2.3. αIIbβ3

αIIbβ3 is the major integrin found on platelets and has been reported to be ectopically expressed in some human tumours and cancer cell lines [110,111]. It has not been observed in HNSCC clinical samples, but has been shown to be expressed in the HSC-3 cell line, where it is involved in transmigration [111], and to mediate the metastasis of nasopharyngeal carcinoma cells through supporting tumour–platelet interactions [112]. Analysis of head and neck cancer molecular alterations and signalling pathways in patient data from The Cancer Genome Atlas identified the αIIb (and α5) integrin subunit genes to be mutated in patients with progressive disease, and the RGD-containing ligands fibrinogen, and vitronectin to be differentially expressed [113]. Further investigation of these pathways may reveal new therapeutic strategies against head and neck cancer progression, particularly since mutations in integrin genes are not commonly reported in cancers.

## 3. Applications of Integrin-Targeted Agents

### 3.1. Imaging

Targeting RGD-binding integrins for tumour imaging has been extensively studied. The majority of probes target αvβ3 (reviewed in [114,115]), although αvβ6 has also been targeted [116,117,118]. The challenges and opportunities posed by the non-selective nature of many RGD-binding peptides and heterogeneity of tumour expression of integrins also been considered [119].

Clinical trials of ^18^F-galacto-RGD as a PET imaging agent to detect tumours have included HNSCC [64,120,121]. Tumour uptake was heterogenous but usually higher than background, allowing detection of most (10/12) primary tumours and some (2/6) lymph node metastases [64], and ^18^F-galacto-RGD was proposed as an imaging agent for use in selection and monitoring of patients treated with antiangiogenic therapies. ^111^In-RGD_2_ has been investigated preclinically and showed promising results in selectively imaging tumour angiogenesis [63].

Non-radioactive imaging modalities based on RGD are also in development. RGD-bound near-IR emitting quantum dots allowed non-invasive imaging of xenografts up to 12 hours after injection, with the best image intensity obtained up to 6 hours after treatment [122]. Other near-IR reagents are being developed to assist with intraoperative tumour detection. Atallah et al. used AngioStamp^TM^ 800, a labelled RGD tetramer, to facilitate removal of residual tumour tissue in an orthotopic HNSCC mouse model. NIR-guided surgery increased the recurrence free survival rate by 50%. This orthotopic model allows long term follow up of mice, but the CAL33 tumour cell line used was highly αvβ3-expressing which does not represent HNSCC clinical integrin expression profiles [123]. The same group has used intraoperative αvβ3 imaging to improve the resection of lymph node metastases [124]. An indocyanine green-labelled αvβ6 antibody has also been proposed to improve resection margins by detecting tumour-specific expression at the invading tumour edge [118].

### 3.2. Targeted Therapeutic Delivery

The overexpression of integrins on tumour cells or associated vasculature can be used to selectively deliver a cytotoxic agent to the tumour, thereby reducing toxicity to normal tissue. The general principles and breadth of integrin-targeted drug delivery have been reviewed extensively elsewhere [125,126,127]. Most commonly peptides containing RGD or similar sequences are used to target integrins. The RGD-based approach is common with imaging; other sequences which require activation before binding such as NGR/iso-DGR [128], or CRGDKGPDC, which promotes tumour uptake through the CendR pathway, have also been used [129].

Specific to targeting HNSCC, Tsien et al. [130,131] have developed a peptide simultaneously targeting RGD-binding integrins using cRGDfC and a MMP-2 activatable peptide, building on their observation that the MMPs are also overexpresssd in HNSCC [132] and interact with αvβ3. cRGDfK and a small molecule αvβ3 antagonist have also been used to deliver gold nanoshells to αvβ3-expressing SCC-4 HNSCC xenografts [133]. Radiolabelled nanoshells were effective imaging agents, but could also be used to deliver therapeutic radioisotopes. The nanoshells were also used for photothermal therapy, resulting in an increase in efficacy over untargeted therapy.

αvβ3 is the most popular target for drug delivery, but it is not the only receptor targeted. Agents have been targeted to β1 using peptide (C_16_)_2_-Glu-C_2_-KSSPHSRNSGSGSGSGSGRGDSP designed to contain both the RGD and PHSRN sequences from fibronectin required for high α5β1 affinity to deliver iron oxide particles containing a photodynamic therapy agent [134,135]. Increased uptake was observed in M4e HNSCC xenografts compared to untargeted nanoparticle, suggesting the approach could be used to increase efficacy and decrease toxicity. Antibodies targeting the non-RGD binding integrin subunit β4 have also been used to deliver iron oxide nanoparticles and chemotherapy drugs to HNSCC tumours [136].

### 3.3. Antitumour and Anti-Metastatic Agents

Since expression of the αv and β1 subunits have been associated with proliferation [53] and metastasis [51,53,54,95,107], αvβ3 [65] and αvβ6 [80,84] associated with proliferation and invasion, targeting one or more of these receptors appears to be an attractive strategy to reduce either tumour growth or dissemination. With the exception of cilengitide, very limited preclinical studies have taken place so far [80,88,107]. The observations that inhibiting αv [55], αvβ3 [74], or β1 [101,102,103] increases sensitivity to irradiation is particularly significant for HNSCC as it suggests integrin-targeted therapy could be used as part of a chemoradiotherapy strategy for high risk disease.

Despite high levels of αvβ6 expression on HNSCC, preclinical and clinical studies of αvβ6 targeted agents have not yet been reported. Inhibitory antibodies and peptides have been developed and are the subject of particular interest in pancreatic cancer [137,138]. Hopefully, success here will encourage their investigation in other cancers. The interaction of αvβ6 targeting with radiotherapy also requires investigation.

Further work will be required to establish other members of the RGD-binding integrin subfamily as drug targets in HNSCC. αvβ1 and αvβ8 are relative unknowns in the anticancer field. αIIbβ3 expression on tumour tissue is controversial. Its expression on platelets is known to be significant in haematogenous metastasis [139,140,141,142,143], however the main route of HNSCC metastasis is through the lymph system, so αIIbβ3 targeting is unlikely to be significant in HNSCC.

## 4. Conclusions

RGD-binding integrins are involved in multiple biological processes that are crucial to both maintenance of body homeostasis and pathological conditions. These include cell proliferation, differentiation, apoptosis adhesion, and migration, all processes involved in cell invasion as well as angiogenesis. In terms of head and neck cancer treatment, the main problems associated with radiotherapy and chemotherapy are the profound toxicities and tumour recurrence after treatment. It is therefore important to identify targets that are highly expressed in tumour tissue but not expressed (or only at low levels) in normal tissue. The evidence to date clearly shows that integrin inhibitors can be considered molecular targeted anticancer therapy. αvβ3 appears most suitable for development of imaging agents to improve identification of margins for surgical resection of tumours. Overexpressed integrins will also be useful for targeted delivery of therapeutic agents. To date, αvβ3 and α5β1 have been targeted but the characteristic αvβ6 overexpression seen in HNSCC tumour cells [80] should also provide an important, highly specific approach.

Although published data on αvβ5 is conflicting (see [58,59,60] and [74]), evidence for αvβ6, αvβ8 and α5β1 indicates their inhibition should reduce tumour growth and metastasis. Investigation of other integrins in the β1 subfamily are still at an early stage; αvβ1, α8β1 (RGD binding) and α7β1, α11β1 (non-RGD-binding) integrins have potential as biomarkers of disease location and progression. The involvement of multiple integrins in the progression of head and neck cancer, and in resistance to radio- and chemotherapies, strongly indicates that improved outcomes for HNSCC patients could result from use of integrin inhibitors as precision medicine in combination with current standard-of-care treatments for high risk or progressive disease.

## Figures and Tables

**Table 1:** The expression of arginine-glycine-aspartate (RGD)-binding integrins in squamous cell carcinoma tissues from the head and neck.

Integrin	SE	SCC	E	S	Ref
αvβ3	−/+	−/+	++	+	[43]
αvβ3		−			[44]
αvβ3	+	−/+			[45]
αvβ3	−/+			+++	[46]
αvβ5	+	++	++	+++	[43]
αvβ5	+	+++			[45]
αvβ5	+	++		++	[44]
αvβ1	+++	+++			[45]
αvβ6	−	++	n/a		[44]
αvβ6	++	+++			[45]
αvβ8	+++	+++			[45]
αIIbβ3					
α5β1	+	++	+	++	[43]
α5β1	−	+++			[47]
α5β1	−/+	++			[48]

Key: SE: squamous epithelium; SCC: squamous cell carcinoma; E: endothelial cells; S: stroma; − negative; −/+ weak; + moderate; ++ strong; +++ very strong; blank not tested.

## Abbreviations

AKT: protein kinase b; cRGDfC: cyclo-Arg-Gly-Asp-d-Phe-Cys; ECM: extracellular matrix; EGFR: epidermal growth factor receptor; EMT: epithelial to mesenchymal transition; FAK: focal adhesion kinase; HNSCC: head and neck squamous cell carcinoma; ILK: integrin-linked kinase; MEK/ERK: mitogen-activated protein kinase/extracellular signal-regulated kinase; MMP: matrix metalloprotease; NGR: Asn-Gly-Arg; NPC: nasopharyngeal carcinoma; PARP: poly ADP ribose polymerase; PHSRN: Pro-His-Ser-Arg-Asn; RGD: arginine-glycine-aspartate; TGF-β: transforming growth factor β; SCC: squamous cell carcinoma.

## References

[1] Torre L.A., Bray F., Siegel R.L., Ferlay J., Lortet-Tieulent J., Jemal A. (2015). Global cancer statistics, 2012. CA Cancer J. Clin..

[2] Global Burden of Disease Cancer Collaboration (2017). Global, Regional, and National Cancer Incidence, Mortality, Years of Life Lost, Years Lived With Disability, and Disability-Adjusted Life-years for 32 Cancer Groups, 1990 to 2015: A Systematic Analysis for the Global Burden of Disease Study. JAMA Oncol..

[3] GBD 2015 Mortality and Causes of Death Collaborators (2016). Global, regional, and national life expectancy, all-cause mortality, and cause-specific mortality for 249 causes of death, 1980–2015: A systematic analysis for the Global Burden of Disease Study 2015. Lancet.

[4] Walden M.J., Aygun N. (2013). Head and neck cancer. Semin. Roentgenol..

[5] Neville B.W., Day T.A. (2002). Oral cancer and precancerous lesions. CA Cancer J. Clin..

[6] Adelstein D.J., Li Y., Adams G.L., Wagner H., Kish J.A., Ensley J.F., Schuller D.E., Forastiere A.A. (2003). An intergroup phase III comparison of standard radiation therapy and two schedules of concurrent chemoradiotherapy in patients with unresectable squamous cell head and neck cancer. J. Clin. Oncol..

[7] Bourhis J., Sire C., Graff P., Gregoire V., Maingon P., Calais G., Gery B., Martin L., Alfonsi M., Desprez P. (2012). Concomitant chemoradiotherapy versus acceleration of radiotherapy with or without concomitant chemotherapy in locally advanced head and neck carcinoma (GORTEC 99-02): An open-label phase 3 randomised trial. Lancet Oncol..

[8] Calais G., Bardet E., Sire C., Alfonsi M., Bourhis J., Rhein B., Tortochaux J., Man Y.T., Auvray H., Garaud P. (2004). Radiotherapy with concomitant weekly docetaxel for Stages III/IV oropharynx carcinoma. Results of the 98–02 GORTEC Phase II trial. Int. J. Radiat. Oncol. Biol. Phys..

[9] Denis F., Garaud P., Bardet E., Alfonsi M., Sire C., Germain T., Bergerot P., Rhein B., Tortochaux J., Calais G. (2004). Final results of the 94–01 French Head and Neck Oncology and Radiotherapy Group randomized trial comparing radiotherapy alone with concomitant radiochemotherapy in advanced-stage oropharynx carcinoma. J. Clin. Oncol..

[10] Ang K.K., Zhang Q., Rosenthal D.I., Nguyen-Tan P.F., Sherman E.J., Weber R.S., Galvin J.M., Bonner J.A., Harris J., El-Naggar A.K. (2014). Randomized phase III trial of concurrent accelerated radiation plus cisplatin with or without cetuximab for stage III to IV head and neck carcinoma: RTOG 0522. J. Clin. Oncol..

[11] Giralt J., Trigo J., Nuyts S., Ozsahin M., Skladowski K., Hatoum G., Daisne J.F., Yunes Ancona A.C., Cmelak A., Mesia R. (2015). Panitumumab plus radiotherapy versus chemoradiotherapy in patients with unresected, locally advanced squamous-cell carcinoma of the head and neck (CONCERT-2): A randomised, controlled, open-label phase 2 trial. Lancet Oncol..

[12] Cox D., Brennan M., Moran N. (2010). Integrins as therapeutic targets: Lessons and opportunities. Nat. Rev. Drug Discov..

[13] Campbell I.D., Humphries M.J. (2011). Integrin structure, activation, and interactions. Cold Spring Harb. Perspect. Biol..

[14] Hohenester E. (2014). Signalling complexes at the cell-matrix interface. Curr. Opin. Struct. Biol..

[15] Shimaoka M., Takagi J., Springer T.A. (2002). Conformational regulation of integrin structure and function. Annu. Rev. Biophys. Biomol. Struct..

[16] Hynes R.O. (2002). Integrins: Bidirectional, allosteric signaling machines. Cell.

[17] Avraamides C.J., Garmy-Susini B., Varner J.A. (2008). Integrins in angiogenesis and lymphangiogenesis. Nat. Rev. Cancer.

[18] Goodman S.L., Picard M. (2012). Integrins as therapeutic targets. Trends Pharmacol. Sci..

[19] Goswami S. (2013). Importance of integrin receptors in the field of pharmaceutical & medical science. ABC.

[20] Millard M., Odde S., Neamati N. (2011). Integrin Targeted Therapeutics. Theranostics.

[21] Ley K., Rivera-Nieves J., Sandborn W.J., Shattil S. (2016). Integrin-based therapeutics: Biological basis, clinical use and new drugs. Nat. Rev. Drug Discov..

[22] Bianconi D., Unseld M., Prager G.W. (2016). Integrins in the Spotlight of Cancer. Int. J. Mol. Sci..

[23] Silva R., D’Amico G., Hodivala-Dilke K.M., Reynolds L.E. (2008). Integrins: The Keys to Unlocking Angiogenesis. Arterioscler. Thromb. Vasc. Biol..

[24] Weis S.M., Cheresh D.A. (2011). Tumor angiogenesis: Molecular pathways and therapeutic targets. Nat. Med..

[25] Atkinson S.J., Ellison T.S., Steri V., Gould E., Robinson S.D. (2014). Redefining the role(s) of endothelial αvβ3-integrin in angiogenesis. Biochem. Soc. Trans..

[26] Worthington J.J., Klementowicz J.E., Travis M.A. (2011). TGFb: A sleeping giant awoken by integrins. Trends Biochem. Sci..

[27] Ozawa A., Sato Y., Imabayashi T., Uemura T., Takagi J., Sekiguchi K. (2016). Molecular Basis of the Ligand-binding Specificity of αvβ8 Integrin. J. Biol. Chem..

[28] Cambier S., Gline S., Mu D., Collins R., Araya J., Dolganov G., Einheber S., Boudreau N., Nishimura S.L. (2005). Integrin αvβ8-mediated activation of transforming growth factor-β by perivascular astrocytes: An angiogenic control switch. Am. J. Pathol..

[29] Bianchi A., Gervasi M.E., Bakin A. (2010). Role of β5-integrin in epithelial-mesenchymal transition in response to TGF-β. Cell Cycle.

[30] Bandyopadhyay A., Raghavan S. (2009). Defining the Role of Integrin αvβ6 in Cancer. Curr. Drug Targets.

[31] Dutta A., Li J., Lu H., Akech J., Pratap J., Wang T., Zerlanko B.J., Fitzgerald T.J., Jiang Z., Birbe R. (2014). Integrin αvβ6 promotes an osteolytic program in cancer cells by upregulating MMP2. Cancer Res..

[32] Lamar J.M., Pumiglia K.M., DiPersio C.M. (2008). An Immortalization-Dependent Switch in Integrin Function Up-regulates MMP-9 to Enhance Tumor Cell Invasion. Cancer Res..

[33] Cantor D.I., Cheruku H.R., Nice E.C., Baker M.S. (2015). Integrin αvβ6 sets the stage for colorectal cancer metastasis. Cancer Metastasis Rev..

[34] Brooks S.A., Lomax-Browne H.J., Carter T.M., Kinch C.E., Hall D.M.S. (2009). Molecular interactions in cancer cell metastasis. Acta Histochem..

[35] Staflin K., Krueger J.S., Hachmann J., Forsyth J.S., Lorger M., Steiniger S.C.J., Mee J., Pop C., Salvesen G.S., Janda K.D. (2010). Targeting activated integrin αvβ3 with patient-derived antibodies impacts late-stage multiorgan metastasis. Clin. Exp. Metastasis.

[36] Van der Horst G., van den Hoogen C., Buijs J.T., Cheung H., Bloys H., Pelger R.C.M., Lorenzon G., Heckmann B., Feyen J., Pujuguet P. (2011). Targeting of av-Integrins in Stem/Progenitor Cells and Supportive Microenvironment Impairs Bone Metastasis in Human Prostate Cancer. Neoplasia.

[37] Sutherland M., Gordon A., Shnyder S.D., Patterson L.H., Sheldrake H.M. (2012). RGD-Binding Integrins in Prostate Cancer: Expression Patterns and Therapeutic Prospects against Bone Metastasis. Cancers (Basel).

[38] Sheldrake H.M., Patterson L.H. (2009). Function and antagonism of β3 integrins in the development of cancer therapy. Curr. Cancer Drug Targets.

[39] Brennan M., Cox D. (2014). The therapeutic potential of I-domain integrins. Adv. Exp. Med. Biol..

[40] Sheldrake H.M., Patterson L.H. (2014). Strategies to inhibit tumor associated integrin receptors: Rationale for dual and multi-antagonists. J. Med. Chem..

[41] Hamidi H., Pietila M., Ivaska J. (2016). The complexity of integrins in cancer and new scopes for therapeutic targeting. Br. J. Cancer.

[42] Georgolios A.K., Batistatou A., Charalabopoulos K. (2005). Integrins in head and neck squamous cell carcinoma (HNSCC): A review of the current literature. Cell Commun. Adhes..

[43] Fabricius E.M., Wildner G.P., Kruse-Boitschenko U., Hoffmeister B., Goodman S.L., Raguse J.D. (2011). Immunohistochemical analysis of integrins αvβ3, αvβ5 and α5β1, and their ligands, fibrinogen, fibronectin, osteopontin and vitronectin, in frozen sections of human oral head and neck squamous cell carcinomas. Exp. Ther. Med..

[44] Jones J., Watt F.M., Speight P.M. (1997). Changes in the expression of αv integrins in oral squamous cell carcinomas. J. Oral. Pathol. Med..

[45] Li F., Liu Y., Kan X., Li Y., Liu M., Lu J.G. (2013). Elevated expression of integrin αv and β5 subunit in laryngeal squamous-cell carcinoma associated with lymphatic metastasis and angiogenesis. Pathol. Res. Pract..

[46] Li P., Liu F., Sun L., Zhao Z., Ding X., Shang D., Xu Z., Sun C. (2011). Chemokine receptor 7 promotes cell migration and adhesion in metastatic squamous cell carcinoma of the head and neck by activating integrin αvβ3. Int. J. Mol. Med..

[47] Vitolo D., Ciocci L., Ferrauti P., Cicerone E., Gallo A., De Vincentiis M., Baroni C.D. (2000). α5 integrin distribution and TGFβ1 gene expression in supraglottic carcinoma: Their role in neoplastic local invasion and metastasis. Head Neck.

[48] Shinohara M., Nakamura S., Sasaki M., Kurahara S., Ikebe T., Harada T., Shirasuna K. (1999). Expression of integrins in squamous cell carcinoma of the oral cavity. Correlations with tumor invasion and metastasis. Am. J. Clin. Pathol..

[49] Brooks P.C. (1994). Requirement of Vascular Integrin alphaVbeta3 for Angiogenesis. Science.

[50] Nisato R.E., Tille J.C., Jonczyk A., Goodman S.L., Pepper M.S. (2003). αvβ3 and αvβ5 integrin antagonists inhibit angiogenesis in vitro. Angiogenesis.

[51] Miller S.E., Veale R.B. (2001). Environmental modulation of α(v), α(2) and β(1) integrin subunit expression in human oesophageal squamous cell carcinomas. Cell. Biol. Int..

[52] Hayashido Y., Kitano H., Sakaue T., Fujii T., Suematsu M., Sakurai S., Okamoto T. (2014). Overexpression of integrin αv facilitates proliferation and invasion of oral squamous cell carcinoma cells via MEK/ERK signaling pathway that is activated by interaction of integrin αvβ8 with type I collagen. Int. J. Oncol..

[53] Lu J.G., Li Y., Li L., Kan X. (2011). Overexpression of osteopontin and integrin αv in laryngeal and hypopharyngeal carcinomas associated with differentiation and metastasis. J. Cancer Res. Clin. Oncol..

[54] Xuan S.-H., Zhou Y.-G., Pan J.-Q., Zhu W., Xu P. (2013). Overexpression of integrin αv in the human nasopharyngeal carcinoma associated with metastasis and progression. Cancer Biomark..

[55] Ou J., Luan W., Deng J., Sa R., Liang H. (2012). αV integrin induces multicellular radioresistance in human nasopharyngeal carcinoma via activating SAPK/JNK pathway. PLoS ONE.

[56] Lu J.G., Sun Y.N., Wang C., Jin de J., Liu M. (2009). Role of the αv-integrin subunit in cell proliferation, apoptosis and tumor metastasis of laryngeal and hypopharyngeal squamous cell carcinomas: A clinical and in vitro investigation. Eur. Arch. Oto-Rhino-Laryngol..

[57] Kurokawa A., Nagata M., Kitamura N., Noman A.A., Ohnishi M., Ohyama T., Kobayashi T., Shingaki S., Takagi R., Oral Maxillofacial Pathology and Surgery Group (2008). Diagnostic value of integrin α3, β4, and β5 gene expression levels for the clinical outcome of tongue squamous cell carcinoma. Cancer.

[58] Stojanovic N., Brozovic A., Majhen D., Bosnar M.H., Fritz G., Osmak M., Ambriovic-Ristov A. (2016). Integrin αvβ3 expression in tongue squamous carcinoma cells Cal27 confers anticancer drug resistance through loss of pSrc(Y418). Biochim. Biophys. Acta.

[59] Jones J., Sugiyama M., Speight P.M., Watt F.M. (1996). Restoration of αvβ5 integrin expression in neoplastic keratinocytes results in increased capacity for terminal differentiation and suppression of anchorage-independent growth. Oncogene.

[60] Janes S.M., Watt F.M. (2004). Switch from αvβ5 to αvβ6 integrin expression protects squamous cell carcinomas from anoikis. J. Cell. Biol..

[61] Brooks P.C., Montgomery A.M., Rosenfeld M., Reisfeld R.A., Hu T., Klier G., Cheresh D.A. (1994). Integrin αvβ3 antagonists promote tumor regression by inducing apoptosis of angiogenic blood vessels. Cell.

[62] Max R., Gerritsen R.R., Nooijen P.T., Goodman S.L., Sutter A., Keilholz U., Ruiter D.J., De Waal R.M. (1997). Immunohistochemical analysis of integrin αvβ3 expression on tumor-associated vessels of human carcinomas. Int. J. Cancer.

[63] Terry S.Y., Abiraj K., Frielink C., van Dijk L.K., Bussink J., Oyen W.J., Boerman O.C. (2014). Imaging integrin αvβ3 on blood vessels with 111In-RGD2 in head and neck tumor xenografts. J. Nucl. Med..

[64] Beer A.J., Grosu A.L., Carlsen J., Kolk A., Sarbia M., Stangier I., Watzlowik P., Wester H.J., Haubner R., Schwaiger M. (2007). [18F]galacto-RGD positron emission tomography for imaging of αvβ3 expression on the neovasculature in patients with squamous cell carcinoma of the head and neck. Clin. Cancer Res..

[65] Qin X., Yan M., Zhang J., Wang X., Shen Z., Lv Z., Li Z., Wei W., Chen W. (2016). TGFβ3-mediated induction of Periostin facilitates head and neck cancer growth and is associated with metastasis. Sci. Rep..

[66] Kumar C.C., Malkowski M., Yin Z., Tanghetti E., Yaremko B., Nechuta T., Varner J., Liu M., Smith E.M., Neustadt B. (2001). Inhibition of angiogenesis and tumor growth by SCH221153, a dual αvβ3 and αvβ5 integrin receptor antagonist. Cancer Res..

[67] Beekman K.W., Colevas A.D., Cooney K., Dipaola R., Dunn R.L., Gross M., Keller E.T., Pienta K.J., Ryan C.J., Smith D. (2006). Phase II evaluations of cilengitide in asymptomatic patients with androgen-independent prostate cancer: Scientific rationale and study design. Clin. Genitourin. Cancer.

[68] Ruffini F., Graziani G., Levati L., Tentori L., D’Atri S., Lacal P.M. (2015). Cilengitide downmodulates invasiveness and vasculogenic mimicry of neuropilin 1 expressing melanoma cells through the inhibition of αvβ5 integrin. Int. J. Cancer.

[69] Stupp R., Hegi M.E., Neyns B., Goldbrunner R., Schlegel U., Clement P.M., Grabenbauer G.G., Ochsenbein A.F., Simon M., Dietrich P.Y. (2010). Phase I/IIa study of cilengitide and temozolomide with concomitant radiotherapy followed by cilengitide and temozolomide maintenance therapy in patients with newly diagnosed glioblastoma. J. Clin. Oncol..

[70] Stupp R., Hegi M.E., Gorlia T., Erridge S.C., Perry J., Hong Y.K., Aldape K.D., Lhermitte B., Pietsch T., Grujicic D. (2014). Cilengitide combined with standard treatment for patients with newly diagnosed glioblastoma with methylated MGMT promoter (CENTRIC EORTC 26071–22072 study): A multicentre, randomised, open-label, phase 3 trial. Lancet Oncol..

[71] Raguse J.D., Gath H.J., Bier J., Riess H., Oettle H. (2004). Cilengitide (EMD 121974) arrests the growth of a heavily pretreated highly vascularised head and neck tumour. Oral Oncol..

[72] Vermorken J.B., Guigay J., Mesia R., Trigo J.M., Keilholz U., Kerber A., Bethe U., Picard M., Brummendorf T.H. (2011). Phase I/II trial of cilengitide with cetuximab, cisplatin and 5-fluorouracil in recurrent and/or metastatic squamous cell cancer of the head and neck: Findings of the phase I part. Br. J. Cancer.

[73] Vermorken J.B., Peyrade F., Krauss J., Mesia R., Remenar E., Gauler T.C., Keilholz U., Delord J.P., Schafhausen P., Erfan J. (2014). Cisplatin, 5-fluorouracil, and cetuximab (PFE) with or without cilengitide in recurrent/metastatic squamous cell carcinoma of the head and neck: Results of the randomized phase I/II ADVANTAGE trial (phase II part). Ann. Oncol..

[74] Heiduschka G., Lill C., Schneider S., Seemann R., Kornek G., Schmid R., Kotowski U., Thurnher D. (2014). The effect of cilengitide in combination with irradiation and chemotherapy in head and neck squamous cell carcinoma cell lines. Strahlenther. Onkol..

[75] Wichmann G., Korner C., Boehm A., Mozet C., Dietz A. (2015). Stimulation by Monocyte Chemoattractant Protein-1 Modulates the Ex Vivo Colony Formation by Head and Neck Squamous Cell Carcinoma Cells. Anticancer Res..

[76] Wichmann G., Cedra S., Schlegel D., Kolb M., Wiegand S., Boehm A., Hofer M., Dietz A. (2017). Cilengitide and Cetuximab Reduce Cytokine Production and Colony Formation of Head and Neck Squamous Cell Carcinoma Cells Ex Vivo. Anticancer Res..

[77] Franovic A., Elliott K.C., Seguin L., Camargo M.F., Weis S.M., Cheresh D.A. (2015). Glioblastomas require integrin αvβ3/PAK4 signaling to escape senescence. Cancer Res..

[78] Becker A., von Richter O., Kovar A., Scheible H., van Lier J.J., Johne A. (2015). Metabolism and disposition of the αv-integrin αvβ3/αvβ5 receptor antagonist cilengitide, a cyclic polypeptide, in humans. J. Clin. Pharmacol..

[79] Cirkel G.A., Milojkovic Kerklaan B., Vanhoutte F., Van der Aa A., Lorenzon G., Namour F., Pujuguet P., Darquenne S., de Vos F.Y.F., Snijders T.J. (2016). A dose escalating phase I study of GLPG0187, a broad spectrum integrin receptor antagonist, in adult patients with progressive high-grade glioma and other advanced solid malignancies. Investig. New Drugs.

[80] Hsiao J.R., Chang Y., Chen Y.L., Hsieh S.H., Hsu K.F., Wang C.F., Tsai S.T., Jin Y.T. (2010). Cyclic αvβ6-targeting peptide selected from biopanning with clinical potential for head and neck squamous cell carcinoma. Head Neck.

[81] Ramos D.M., Chen B.L., Boylen K., Stern M., Kramer R.H., Sheppard D., Nishimura S.L., Greenspan D., Zardi L., Pytela R. (1997). Stromal fibroblasts influence oral squamous-cell carcinoma cell interactions with tenascin-C. Int. J. Cancer.

[82] Eriksen J.G., Steiniche T., Sogaard H., Overgaard J. (2004). Expression of integrins and E-cadherin in squamous cell carcinomas of the head and neck. APMIS.

[83] Liu S., Liang B., Gao H., Zhang F., Wang B., Dong X., Niu J. (2013). Integrin αvβ6 as a novel marker for diagnosis and metastatic potential of thyroid carcinoma. Head Neck Oncol..

[84] Ramos D.M., But M., Regezi J., Schmidt B.L., Atakilit A., Dang D., Ellis D., Jordan R., Li X. (2002). Expression of integrin β6 enhances invasive behavior in oral squamous cell carcinoma. Matrix Biol..

[85] Larjava H., Haapasalmi K., Salo T., Wiebe C., Uitto V.J. (1996). Keratinocyte integrins in wound healing and chronic inflammation of the human periodontium. Oral Dis..

[86] Lee C., Lee C., Lee S., Siu A., Ramos D.M. (2014). The cytoplasmic extension of the integrin β6 subunit regulates epithelial-to-mesenchymal transition. Anticancer Res..

[87] Ramos D.M., Dang D., Sadler S. (2009). The role of the integrin αvβ6 in regulating the epithelial to mesenchymal transition in oral cancer. Anticancer Res..

[88] Xue H., Atakilit A., Zhu W., Li X., Ramos D.M., Pytela R. (2001). Role of the αvβ6 integrin in human oral squamous cell carcinoma growth in vivo and in vitro. Biochem. Biophys. Res. Commun..

[89] Koivisto L., Grenman R., Heino J., Larjava H. (2000). Integrins α5β1, αvβ1, and αvβ6collaborate in squamous carcinoma cell spreading and migration on fibronectin. Exp. Cell Res..

[90] Reed N.I., Tang Y.Z., McIntosh J., Wu Y., Molnar K.S., Civitavecchia A., Sheppard D., DeGrado W.F., Jo H. (2016). Exploring N-Arylsulfonyl-l-proline Scaffold as a Platform for Potent and Selective αvβ1 Integrin Inhibitors. ACS Med. Chem. Lett..

[91] Reed N.I., Jo H., Chen C., Tsujino K., Arnold T.D., DeGrado W.F., Sheppard D. (2015). The αvβ1 integrin plays a critical in vivo role in tissue fibrosis. Sci. Transl. Med..

[92] Adams J., Anderson E.C., Blackham E.E., Chiu Y.W.R., Clarke T., Eccles N., Gill L.A., Haye J.J., Haywood H.T., Hoenig C.R. (2014). Structure Activity Relationships of αv Integrin Antagonists for Pulmonary Fibrosis by Variation in Aryl Substituents. ACS Med. Chem. Lett..

[93] Goodman S.L., Holzemann G., Sulyok G.A.G., Kessler H. (2002). Nanomolar Small Molecule Inhibitors for αnβ6, αnβ5, and αnβ3 Integrins. J. Med. Chem..

[94] Ni R., Shen X., Wu H., Zhu W., Ni J., Huang Z., Song Y., Gao X. (2010). Expression and significance of integrins subunits in laryngeal squamous cell carcinoma. Lin Chuang Er Bi Yan Hou Tou Jing Wai Ke Za Zhi.

[95] Wang D., Muller S., Amin A.R., Huang D., Su L., Hu Z., Rahman M.A., Nannapaneni S., Koenig L., Chen Z. (2012). The pivotal role of integrin β1 in metastasis of head and neck squamous cell carcinoma. Clin. Cancer Res..

[96] De Moraes F.P., Lourenço S.V., Ianez R.C., de Sousa E.A., Silva M.M., Damascena A.S., Kowalski L.P., Soares F.A., Coutinho-Camillo C.M. (2017). Expression of stem cell markers in oral cavity and oropharynx squamous cell carcinoma. Oral Surg. Oral Med. Oral Pathol. Oral Radiol..

[97] Yen Y.C., Hsiao J.R., Jiang S.S., Chang J.S., Wang S.H., Shen Y.Y., Chen C.H., Chang I.S., Chang J.Y., Chen Y.W. (2015). Insulin-like growth factor-independent insulin-like growth factor binding protein 3 promotes cell migration and lymph node metastasis of oral squamous cell carcinoma cells by requirement of integrin β1. Oncotarget.

[98] Koukourakis M.I., Giatromanolaki A., Tsakmaki V., Danielidis V., Sivridis E. (2012). Cancer stem cell phenotype relates to radio-chemotherapy outcome in locally advanced squamous cell head-neck cancer. Br. J. Cancer.

[99] Bragado P., Estrada Y., Sosa M.S., Avivar-Valderas A., Cannan D., Genden E., Teng M., Ranganathan A.C., Wen H.C., Kapoor A. (2012). Analysis of marker-defined HNSCC subpopulations reveals a dynamic regulation of tumor initiating properties. PLoS ONE.

[100] Ming X.Y., Fu L., Zhang L.Y., Qin Y.R., Cao T.T., Chan K.W., Ma S., Xie D., Guan X.Y. (2016). Integrin α7 is a functional cancer stem cell surface marker in oesophageal squamous cell carcinoma. Nat. Commun..

[101] Eke I., Deuse Y., Hehlgans S., Gurtner K., Krause M., Baumann M., Shevchenko A., Sandfort V., Cordes N. (2012). β(1)Integrin/FAK/cortactin signaling is essential for human head and neck cancer resistance to radiotherapy. J. Clin. Investig..

[102] Dickreuter E., Eke I., Krause M., Borgmann K., van Vugt M.A., Cordes N. (2016). Targeting of β1 integrins impairs DNA repair for radiosensitization of head and neck cancer cells. Oncogene.

[103] Eke I., Zscheppang K., Dickreuter E., Hickmann L., Mazzeo E., Unger K., Krause M., Cordes N. (2015). Simultaneous β1 integrin-EGFR targeting and radiosensitization of human head and neck cancer. J. Natl. Cancer Inst..

[104] Parajuli H., Teh M.T., Abrahamsen S., Christoffersen I., Neppelberg E., Lybak S., Osman T., Johannessen A.C., Gullberg D., Skarstein K. (2016). Integrin α11 is overexpressed by tumour stroma of head and neck squamous cell carcinoma and correlates positively with alpha smooth muscle actin expression. J. Oral Pathol. Med..

[105] Steglich A., Vehlow A., Eke I., Cordes N. (2015). α integrin targeting for radiosensitization of three-dimensionally grown human head and neck squamous cell carcinoma cells. Cancer Lett..

[106] Xie J.J., Guo J.C., Wu Z.Y., Xu X.E., Wu J.Y., Chen B., Ran L.Q., Liao L.D., Li E.M., Xu L.Y. (2016). Integrin α5 promotes tumor progression and is an independent unfavorable prognostic factor in esophageal squamous cell carcinoma. Hum. Pathol..

[107] Jung A.C., Ray A.M., Ramolu L., Macabre C., Simon F., Noulet F., Blandin A.F., Renner G., Lehmann M., Choulier L. (2015). Caveolin-1-negative head and neck squamous cell carcinoma primary tumors display increased epithelial to mesenchymal transition and prometastatic properties. Oncotarget.

[108] Cianfrocca M.E., Kimmel K.A., Gallo J., Cardoso T., Brown M.M., Hudes G., Lewis N., Weiner L., Lam G.N., Brown S.C. (2006). Phase 1 Trial of the Antiangiogenic Peptide ATN-161 (Ac-PHSCN-NH2), a beta Integrin Antagonist, in Patients with Solid Tumours. Br. J. Cancer.

[109] Barkan D., Chambers A.F. (2011). β1-integrin: A Potential Therapeutic Target in the Battle against Cancer Recurrence. Clin. Cancer Res..

[110] Lonsdorf A.S., Kramer B.F., Fahrleitner M., Schonberger T., Gnerlich S., Ring S., Gehring S., Schneider S.W., Kruhlak M.J., Meuth S.G. (2012). Engagement of αIIbβ3 (GPIIb/IIIa) with αvβ3 integrin mediates interaction of melanoma cells with platelets: A connection to hematogenous metastasis. J. Biol. Chem..

[111] Parikka M., Nissinen L., Kainulainen T., Bruckner-Tuderman L., Salo T., Heino J., Tasanen K. (2006). Collagen XVII promotes integrin-mediated squamous cell carcinoma transmigration—A novel role for αIIβ integrin and tirofiban. Exp. Cell Res..

[112] Chen Y.W., Chen J.K., Wang J.S. (2009). Exercise affects platelet-promoted tumor cell adhesion and invasion to endothelium. Eur. J. Appl. Physiol..

[113] Bornstein S., Schmidt M., Choonoo G., Levin T., Gray J., Thomas C.R., Wong M., McWeeney S. (2016). IL-10 and integrin signaling pathways are associated with head and neck cancer progression. BMC Genom..

[114] Liu Z., Wang F. (2013). Development of RGD-based radiotracers for tumor imaging and therapy: Translating from bench to bedside. Curr. Mol. Med..

[115] Liu Z., Yu L., Wang X., Zhang X., Liu M., Zeng W. (2016). Integrin (αvβ3) Targeted RGD Peptide Based Probe for Cancer Optical Imaging. Curr. Protein Pept. Sci..

[116] Liu H., Wu Y., Wang F., Liu Z. (2014). Molecular imaging of integrin αvβ6 expression in living subjects. Am. J. Nucl. Med. Mol. Imaging.

[117] Nothelfer E.M., Zitzmann-Kolbe S., Garcia-Boy R., Kramer S., Herold-Mende C., Altmann A., Eisenhut M., Mier W., Haberkorn U. (2009). Identification and characterization of a peptide with affinity to head and neck cancer. J. Nucl. Med..

[118] Zhang C., Zhang Y., Hong K., Zhu S., Wan J. (2017). Photoacoustic and Fluorescence Imaging of Cutaneous Squamous Cell Carcinoma in Living Subjects Using a Probe Targeting Integrin αvβ6. Sci. Rep..

[119] Liu S. (2015). Radiolabeled Cyclic RGD Peptide Bioconjugates as Radiotracers Targeting Multiple Integrins. Bioconj. Chem..

[120] Beer A.J., Haubner R., Sarbia M., Goebel M., Luderschmidt S., Grosu A.L., Schnell O., Niemeyer M., Kessler H., Wester H.J. (2006). Positron emission tomography using [18F]Galacto-RGD identifies the level of integrin αvβ3 expression in man. Clin. Cancer Res..

[121] Beer A.J., Lorenzen S., Metz S., Herrmann K., Watzlowik P., Wester H.J., Peschel C., Lordick F., Schwaiger M. (2008). Comparison of integrin αvβ3 expression and glucose metabolism in primary and metastatic lesions in cancer patients: A PET study using 18F-galacto-RGD and 18F-FDG. J. Nucl. Med..

[122] Huang H., Bai Y.L., Yang K., Tang H., Wang Y.W. (2013). Optical imaging of head and neck squamous cell carcinoma in vivo using arginine-glycine-aspartic acid peptide conjugated near-infrared quantum dots. OncoTargets Ther.

[123] Atallah I., Milet C., Henry M., Josserand V., Reyt E., Coll J.L., Hurbin A., Righini C. (2016). Near-infrared fluorescence imaging-guided surgery improves recurrence-free survival rate in novel orthotopic animal model of head and neck squamous cell carcinoma. Head Neck.

[124] Atallah I., Milet C., Quatre R., Henry M., Reyt E., Coll J.L., Hurbin A., Righini C.A. (2015). Role of near-infrared fluorescence imaging in the resection of metastatic lymph nodes in an optimized orthotopic animal model of HNSCC. Eur. Ann. Oto-Rhino-Laryngol. Head Neck Dis..

[125] Arosio D., Casagrande C. (2016). Advancement in integrin facilitated drug delivery. Adv. Drug Deliv. Rev..

[126] Katsamakas S., Chatzisideri T., Thysiadis S., Sarli V. (2017). RGD-mediated delivery of small-molecule drugs. Future Med. Chem..

[127] Dissanayake S., Denny W.A., Gamage S., Sarojini V. (2017). Recent developments in anticancer drug delivery using cell penetrating and tumor targeting peptides. J. Control. Release.

[128] Spitaleri A., Mari S., Curnis F., Traversari C., Longhi R., Bordignon C., Corti A., Rizzardi G.-P., Musco G. (2008). Structural basis for the interaction of isoDGR with the RGD-binding site of αnβ3 integrin. J. Biol. Chem..

[129] Tambet T., Sugahara K.N., Ruoslahti E. (2013). Tumor-penetrating peptides. Front. Oncol..

[130] Crisp J.L., Savariar E.N., Glasgow H.L., Ellies L.G., Whitney M.A., Tsien R.Y. (2014). Dual targeting of integrin αvβ3 and matrix metalloproteinase-2 for optical imaging of tumors and chemotherapeutic delivery. Mol. Cancer Ther..

[131] Buckel L., Savariar E.N., Crisp J.L., Jones K.A., Hicks A.M., Scanderbeg D.J., Nguyen Q.T., Sicklick J.K., Lowy A.M., Tsien R.Y. (2015). Tumor radiosensitization by monomethyl auristatin E: Mechanism of action and targeted delivery. Cancer Res..

[132] Hauff S.J., Raju S.C., Orosco R.K., Gross A.M., Diaz-Perez J.A., Savariar E., Nashi N., Hasselman J., Whitney M., Myers J.N. (2014). Matrix-metalloproteinases in head and neck carcinoma-cancer genome atlas analysis and fluorescence imaging in mice. Otolaryngol. Head Neck Surg..

[133] Xie H., Diagaradjane P., Deorukhkar A.A., Goins B., Bao A., Phillips W.T., Wang Z., Schwartz J., Krishnan S. (2011). Integrin αvβ3-targeted gold nanoshells augment tumor vasculature-specific imaging and therapy. Int. J. Nanomed..

[134] Wang D., Fei B., Halig L.V., Qin X., Hu Z., Xu H., Wang Y.A., Chen Z., Kim S., Shin D.M. (2014). Targeted iron-oxide nanoparticle for photodynamic therapy and imaging of head and neck cancer. ACS Nano.

[135] Halig L.V., Wang D., Wang A.Y., Chen Z.G., Fei B. (2013). Biodistribution Study of Nanoparticle Encapsulated Photodynamic Therapy Drugs Using Multispectral Imaging. Proc. SPIE Int. Soc. Opt. Eng..

[136] Kim D.H., Vitol E.A., Liu J., Balasubramanian S., Gosztola D.J., Cohen E.E., Novosad V., Rozhkova E.A. (2013). Stimuli-responsive magnetic nanomicelles as multifunctional heat and cargo delivery vehicles. Langmuir.

[137] Eberlein C., Kendrew J., McDaid K., Alfred A., Kang J.S., Jacobs V.N., Ross S.J., Rooney C., Smith N.R., Rinkenberger J. (2013). A human monoclonal antibody 264RAD targeting αvβ6 integrin reduces tumour growth and metastasis, and modulates key biomarkers in vivo. Oncogene.

[138] Howard M.J., Dicara D., Marshall J.F. (2006). αvβ6 Peptide Ligands and Their Uses. U.S. Patent.

[139] Bakewell S.J., Nestor P., Prasad S., Tomasson M.H., Dowland N., Mehrotra M., Scarborough R.M., Kanter J., Abe K., Phillips D. (2003). Platelet and Osteoclast β3 Integrins are Critical for Bone Metastasis. Proc. Natl. Acad. Sci. USA.

[140] Trikha M., Zhou Z., Timar J., Raso E., Kennel M., Emmell E., Nakada M. (2002). Multiple Roles for Platelet GPIIb/IIIa and αvβ3 Integrins in Tumor Growth, Angiogenesis, and Metastasis. Cancer Res..

[141] Buergy D., WEnz F., Groden C., Brockmann M.A. (2012). Tumor-platelet interaction in solid tumors. Int. J. Cancer.

[142] Weber M.R., Zuka M., Lorger M., Tschan M., Torbett B.E., Zijlstra A., Quigley J.P., Staflin K., Eliceiri B.P., Krueger J.S. (2016). Activated tumor cell integrin αvβ3 cooperates with platelets to promote extravasation and metastasis from the blood stream. Thromb. Res..

[143] Echtler K., Konrad I., Lorenz M., Schneider S., Hofmaier S., Plenagl F., Stark K., Czermak T., Tirniceriu A., Eichhorn M. (2017). Platelet GPIIb supports initial pulmonary retention but inhibits subsequent proliferation of melanoma cells during hematogenic metastasis. PLoS ONE.

